# Shotgun proteomics data on the impact of hyperglycaemia on platelet protein acetylation by aspirin

**DOI:** 10.1016/j.dib.2018.11.082

**Published:** 2018-11-23

**Authors:** Francesco Finamore, Jean-Luc Reny, Sarah Malacarne, Pierre Fontana, Jean-Charles Sanchez

**Affiliations:** a Translational Biomarker Group, Faculty of Medicine, University of Geneva, Geneva, Switzerland; b Division of Internal Medicine and Rehabilitation, Geneva University Hospitals, Geneva, Switzerland; c Geneva Platelet Group, Faculty of Medicine, University of Geneva, Geneva, Switzerland; d Endocrinology, Diabetology and Nutrition Unit, Geneva University Hospitals, Geneva, Switzerland; e Division of Angiology and Haemostasis, Geneva University Hospitals, Geneva, Switzerland

## Abstract

This data article associated with the manuscript “A high glucose levels is associated with decreased aspirin-mediated acetylation of platelet cyclooxygenase (COX)-1 at serine 529: a pilot study” (Finamore et al., 2018) refers to the shotgun proteomics approach carried out on platelet protein extracts from diabetic patients and healthy controls. Platelet proteins were *in vitro* incubated with 500 µM aspirin for 30 min at 37 °C to enhance the acetylation process. After protein digestion with trypsin, DDA data were acquired on a Thermo QExactive plus using 3 technical replicate injections per sample. Here, we were able to elucidate the preferential sites of aspirin-induced acetylation on a significant fraction of the platelet proteome and to quantify the impact of diabetes on the effect of aspirin on several platelet proteins. Data are available via ProteomeXchange with identifier PXD011582.

**Specifications table**

**Table:** 

Subject area	*Biology, Biochemistry.*
More specific subject area	*Proteomics, Mass spectrometry, Bioinformatics.*
Type of data	*Table, graph, figure.*
How data were acquired	*Mass spectrometry, DDA method using a Thermo QExactive plus.*
Data format	*Raw, filtered, analysed.*
Experimental factors	*Protein extraction and digestion for shotgun proteomics.*
Experimental features	*DDA Mass spectrometry method coupled to label-free quantitation.*
Data source location	*University of Geneva, Geneva, Switzerland.*
Data accessibility	*Data are with this article and the DDA raw files of the case study have been deposited to the ProteomeXchange Consortium via the PRIDE repository with the data identifier*PXD011582*and 10.6019/PXD011582.*
Related research article	*Finamore et al. A high glucose level is associated with decreased aspirin-mediated acetylation of platelet cyclooxygenase (COX)-1 at serine 529: A pilot study.* J Proteomics. 2018 Sep 18. pii: S1874–3919(18)30348-8 [7]

**Value of the data**

• This data article represents one of the first proteomic proofs deciphering the influence of diabetes on aspirin effect on platelet proteins.
• A comprehensive proteomic analysis on aspirin-mediated acetylation of platelet proteins involved in platelet hyper-reactivity useful as a knowledgebase of platelet biology in diabetes.
• The data may offer a compelling invitation to other clinical studies aimed at determining the effect of *in vivo* aspirin administration in diabetes.

## 1 Data

The main aim of this qualitative/quantitative analysis was to characterize the acetylation profile of those platelet proteins that are target of aspirin, beyond COX-1 [1]. The level of aspirin-mediated acetylation was assessed on platelet protein extracts from healthy controls (HbA1c < 6) and diabetic patients (HbA1c > 8) treated *ex vivo* with 500 µM aspirin for 30 min.

**Fig. 1:**
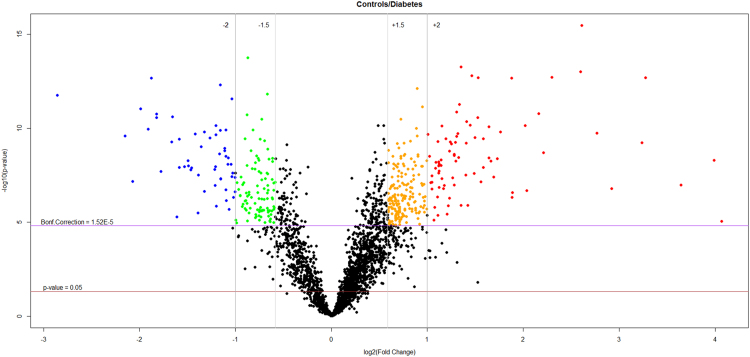
Volcano plot showing the differentially acetylated platelet protein sites in healthy subjects compared to diabetic patients. In orange and in red are those sites with an acetylation level higher than 1.5 and 2, respectively (diabetes reduces their acetylation). In green and blue are those sites with an acetylation level lower than −1.5 and −2, respectively.

A total of 1937 acetylated proteins were identified among the two groups (Supplementary table 1) and 1088 were used for label-free quantitation after filtering features (Rt, *m/z* pairs) for their abundance (≥10^4^), their match with MS/MS spectra (≥1), their reproducibility across replicates (CV ≤ 30%) and their significant variation between groups (p ≤ 0.05) (Supplementary table 2). From the 3292 acetylated residues quantified in this study, the 3.5% and 11.6% showed a 2- and 1.5 fold significant decrease respectively in their acetylation level in diabetes compared to control. We further observed that the 2% and the 5.6% evidenced a 2- and 1.5 fold significant increase of their acetylation state in diabetic patients with respect to controls (Fig. 1).

**Fig. 2:**
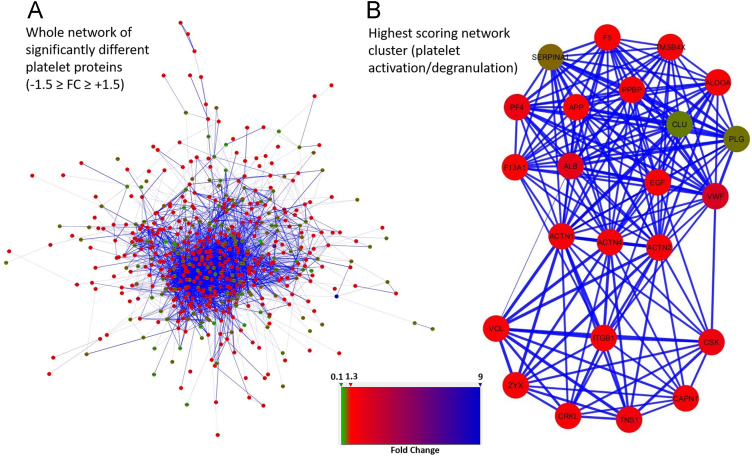
Functional network of protein-protein interaction generated using the STRING database. Nodes represent the differentially acetylated platelet proteins and the colours indicate different acetylation levels, in red the acetylation level is higher in controls than in diabetes, in green the acetylation is higher in diabetes than in controls (A). The highest scoring sub-network extracted from the global one shows the interconnection of proteins involved in platelet activation and degranulation (B).

**Fig. 3:**
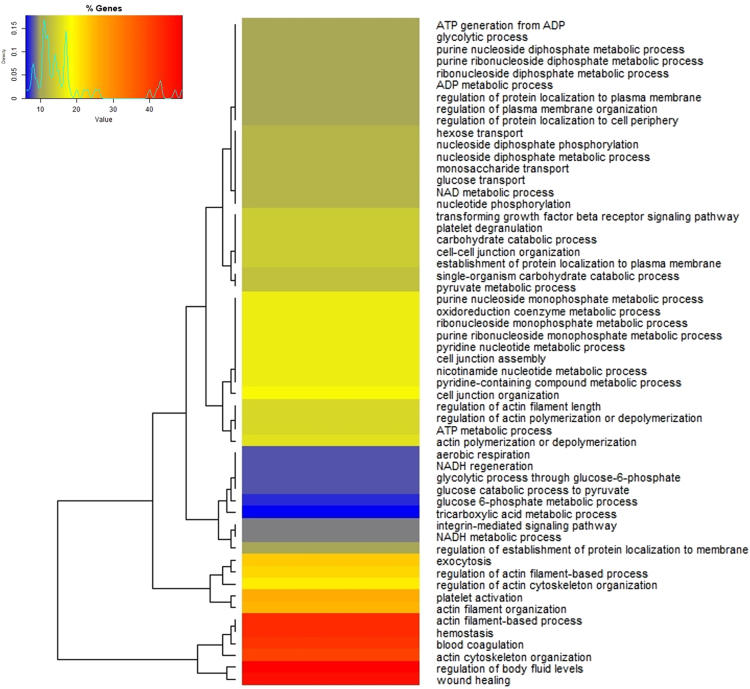
Heat map of the most significant (*p* < 0.01) biological processes obtained from the GO analysis of the differentially acetylated proteins. The number of genes for each GO term are color-coded depending on the degree of gene enrichment in each functional network.

**Fig. 4:**
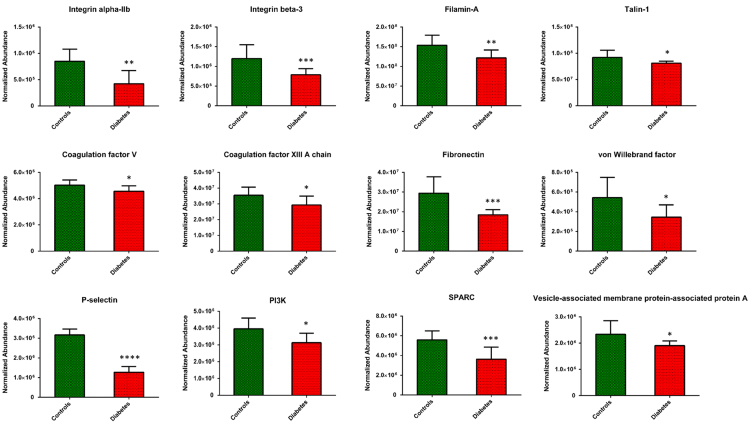
Platelet proteins found differentially expressed in diabetes compared to controls and involved in platelet activation pathway. Average abundances were displayed with standard deviations. Asterisks indicate significance levels: * *p* < .05, ** *p* < .01, *** *p* < .005, **** *p* < .001.

All the proteins containing the significantly different acetylation sites with a fold change higher and lower than ±1.5 were used to generate a functional protein-protein interaction network (Fig. 2A) and finally to perform a GO term enrichment. As revealed by the most significant terms for each functionally grouped network, “*blood coagulation”*, “*haemostasis”* and “*platelet activation”* represented the most significant (*p* ≤ 1.1E^−10^) biological processes with the highest number of genes, as evidenced by the color-coded heat map (Fig. 3) and by the highest scoring sub-network extracted from the global one (Fig. 2B). Among the different proteins involved in those functional pathways we found several classes of integrin receptors (integrin α-2, α-5, α-6, integrin β-3, integrin β-1), structural proteins involved in cell shape change (filamin, talin), coagulation factors (V and XIII), mediator of platelet activation (fibrinogen, von Willebrand factor, P-selectin) [2], [3], [4], degranulation (VAMP, SPARC) [5] and enzymes that trigger platelet aggregation including thromboxane-A synthase and PI3K [6] (Fig. 4). Five different COX-1 sites (Lys 168, Lys 221, Lys 252, Lys 565 and Lys 572) were quantified and 3 out of 5 acetylated residues were also detected by PRM [7], while no information on the critical site of Ser 529 was obtained with this method.

## 2 Experimental design, material and methods

### 2.1 LC-MS/MS analysis, identification and label-free quantitation of the platelet acetylome

Data-dependent acquisition (DDA) was performed on a Q Exactive Plus (ThermoFisher, San Jose, CA) mass spectrometer equipped with a Thermo EASY-nLC coupled with an EASY-Spray source operating at 1.8 kV in positive ion mode. Peptides were trapped at on a 2 cm × 75 µm i.d., PepMap C_18_ precolumn packed with 3 µm particles and 100 Å pore size. Then, separation was achieved in a 50 cm × 75 µm i.d., PepMap C_18_ column packed with 2 µm, 100 Å particles and heated at 50 °C. Liquid chromatography was performed using a 120 min gradient at a flow rate of 250 nL/min using as mobile phase A 0.1% (v/v) formic acid (Fluka) in HPLC-grade water (CHROMASOLV®) and as mobile phase B 0.1% (v/v) formic acid in HPLC-grade acetonitrile (CHROMASOLV®). The gradient program was as follows: 5% B at 5 min; slow ramping to 28% B in 105 min followed by a higher slope increase up to 40% B in 15 min; rapid ramping to 95% B over 10 min and washing column for 15 min. The column was re-equilibrated to 5% B for 24 min after each run. All peptides were analysed with a data-dependent tandem mass spectrometry method that relies on a MS survey scan from which a maximum of 15 precursor ions were selected with an isolation window of 1.6 *m/z* for subsequent fragmentation (normalized collision energy 27%). MS survey scans were acquired at a resolving power of 70,000 in profile mode, whereas MS/MS data were acquired at a resolving power of 17,500 in centroid mode. A dynamic exclusion of 30 s was enabled and precursor ions of charge state 1+ were excluded from data-dependent selection. To identify peptides, raw data were first converted to .mgf file using msConvert tool [8] and the Mascot algorithm (Version 2.5, MatrixScience) was used to query all the MS/MS spectra against the UniProtKB/Swiss-Prot database (Release March 2015; 547,964 sequences) in which we preferentially select the human organism (20.203 sequences) as taxonomy. For database search we selected trypsin as endoprotease with a maximum of four missed tryptic cleavages; a 10 and 6 ppm error tolerance were used for precursor and fragment ion masses, respectively; carbamidomethylation of cysteine residues was selected as fixed modification while methionine oxidation and acetylation at lysine, serine, threonine, tyrosine and N-terminal residues were selected as variable modifications. False discovery rate (FDR) was derived by searching the same MS/MS data against a decoy database composed of reversed protein sequences. The estimated number of false positive peptide identifications was then used to adjust and filter the true positive matches according to an FDR ≤ 1%. Label-free quantitation was performed using Nonlinear Dynamics’ Progenesis QI software, as described elsewhere [9]. Briefly, after run alignment and features extraction, *m/z* and retention time (Rt) pairs were associated to their relative peptide sequence and peak abundances were extracted by integrating the area under the peak curve. Raw abundances were then normalized to a global scaling factor derived from the log space distribution of the ratios between each run and a reference run.

### 2.2 Statistical analysis

Statistical significance between healthy subject and diabetic patient groups was calculated using a Wilcoxon (Mann–Whitney) rank sum test due to the not normally distributed dataset. *P*-values were then corrected using a Holm–Bonferroni method as post hoc correction. Differences in protein abundances were considered significant with a *p*-value less than 0.05 between the two groups.

### 2.3 Functional networks and Gene ontology (GO) Term analysis

Functional networks based on protein-protein interaction were created with the STRING [10] database (version 1.0.4) using the Cytoscape [11] plug-in MCODE [12] and applying a confidence score cut-off of 0.6 to extract the highly interconnected regions of the network (clusters). Gene Ontology (GO) analysis was performed using the Cytoscape plug-in ClueGO [13] (updated 09.02.2016). GO parameters used for functional grouping pathways were: a *p*-value ≤ 0.01 integrated with a Bonferroni step down correction, a GO tree interval between 4 and 8, a minimum number of genes per cluster of 5 with a 5% of genes, a kappa score of 0.4 and an initial group size of 3 terms with a percentage of overlapping terms per group of 50%. Most significant (*p*-value < 0.01) GO terms were represented in a heat map displaying those ones with a higher gene enrichment.

### 2.4 Data availability

The mass spectrometry proteomics data have been deposited to the ProteomeXchange via the PRIDE [14] partner repository with the dataset identifier PXD011582. Figures and tables are with this data article.
